# Serial fibrosis-4 variability and longitudinal patterns are associated with incident heart failure in type 2 diabetes: a 24-month landmark cohort study

**DOI:** 10.3389/fendo.2026.1963810

**Published:** 2026-10-15

**Authors:** Attila Csaba Nagy, Amr Sayed Ghanem, Battamir Ulambayar, Marianna Móré, Mariann Harangi

**Affiliations:** 1 Department of Epidemiology, Faculty of Health Sciences, University of Debrecen, Debrecen, Hungary; 2 Department of Integrative Health Sciences, Institute of Health Sciences, Faculty of Health Sciences, University of Debrecen, Debrecen, Hungary; 3 Division of Metabolism, Department of Internal Medicine, Faculty of Medicine, University of Debrecen, Debrecen, Hungary; 4 Institute of Health Studies, Faculty of Health Sciences, University of Debrecen, Debrecen, Hungary; 5 Eötvös Loránd Research Network–University of Debrecen (ELKH-UD) Vascular Pathophysiology Research Group 11003, University of Debrecen, Debrecen, Hungary

**Keywords:** FIB-4 index, heart failure, landmark analysis, longitudinal biomarkers, real-world data, trajectory patterns, type 2 diabetes mellitus

## Abstract

**Background:**

The prognostic relevance of serial Fibrosis-4 index (FIB-4) changes for incident heart failure (HF) in type 2 diabetes mellitus (T2DM) remains unclear. We examined whether within-person FIB-4 variability and longitudinal patterns were associated with HF after explicit adjustment for chronological age.

**Methods:**

Routinely collected clinical data from 2000 to 2025 were analyzed using a 24-month landmark design. FIB-4 measurements from months 0 to 18 defined the exposure, followed by a 6-month lag before HF follow-up. Variability was calculated as the within-person standard deviation of log_2_-transformed FIB-4. Using a threshold of 2.67, patients were classified as never high, improving from high, worsening to high, or persistently high. Associations with incident HF were estimated using multivariable Cox regression.

**Results:**

Among 8,221 patients, 1,811 developed HF during 50,640 person-years. Greater FIB-4 variability was associated with a higher HF hazard (adjusted hazard ratio [HR], 1.73; 95% confidence interval [CI], 1.43–2.09). Compared with the never-high pattern, persistent-high FIB-4 (HR, 1.73; 95% CI, 1.35–2.20) and worsening to high (HR, 1.28; 95% CI, 1.01–1.62) were associated with higher HF hazards, whereas improving from high was not (HR, 0.95; 95% CI, 0.70–1.28). Adding serial FIB-4 features resulted in a small, statistically uncertain improvement in temporal-validation discrimination (ΔC-index, 0.0069; 95% CI, −0.0043 to 0.0190).

**Discussion:**

Serial FIB-4 variability and persistent elevation were associated with incident HF independently of chronological age. These measures may characterize longitudinal multisystem cardiometabolic burden, but their incremental predictive value was limited. External validation is required before clinical application.

## Introduction

Type 2 diabetes mellitus (T2DM) is a major and growing public health concern worldwide, driven partly by population aging, physical inactivity, and obesity (1). Cardiovascular disease (CVD) remains the predominant cause of morbidity and mortality among people living with T2DM (2). Compared with individuals without diabetes, patients with T2DM experience a substantially increased risk of cardiovascular complications (3). In addition to atherosclerotic events, heart failure (HF) represents an important clinical manifestation of cardiovascular disease in T2DM and contributes to hospitalization, functional impairment, and adverse long-term outcomes.

The development of cardiovascular complications in T2DM is multifactorial and reflects a complex interaction among chronic hyperglycemia, insulin resistance, lipid abnormalities, chronic low-grade inflammation, endothelial dysfunction, hypertension, and chronic kidney disease (2, 4). These interrelated abnormalities may promote microvascular dysfunction, myocardial remodeling, fibrosis, and progressive impairment of cardiac function. Although established factors such as age, sex, smoking, and blood pressure are commonly incorporated into cardiovascular risk assessment (5), routinely available laboratory parameters may provide additional information on the cumulative systemic burden associated with T2DM (6).

Liver dysfunction may represent an additional component of the pathophysiological transition from T2DM to cardiovascular disease. Metabolic dysfunction-associated steatotic liver disease (MASLD) is highly prevalent among patients with T2DM and is associated with increased cardiovascular morbidity and mortality (7). Hepatic steatosis and fibrosis share several pathophysiological pathways with HF, including insulin resistance, chronic inflammation, oxidative stress, endothelial dysfunction, and altered lipid metabolism. Conversely, subclinical cardiac dysfunction and venous congestion may affect liver-related laboratory measurements. Associations between hepatic abnormalities and HF may therefore reflect a bidirectional cardiorenal–hepatic relationship and the broader burden of multisystem cardiometabolic disease rather than isolated liver injury.

The Fibrosis-4 (FIB-4) index is a simple, non-invasive fibrosis score calculated from age, aspartate aminotransferase, alanine aminotransferase, and platelet count (8). Beyond its established hepatic application, FIB-4 may reflect the combined influence of metabolic, inflammatory, hematological, and age-related processes. Previous studies have reported associations between elevated FIB-4 and cardiovascular disease, cardiovascular mortality, and other adverse outcomes among patients with diabetes (9, 10). However, most previous studies have evaluated FIB-4 using a single measurement or conventional baseline risk categories. Such approaches do not capture within-person variability, persistent elevation, or transitions between lower and higher FIB-4 categories over time.

The relationship between serial FIB-4 characteristics and incident HF remains insufficiently characterized in patients with T2DM. This question is also methodologically important because chronological age is a direct component of the FIB-4 formula and, independently, a major determinant of HF risk. Consequently, associations between FIB-4 and HF may be substantially confounded if age is not explicitly controlled. Furthermore, longitudinal evidence on serial FIB-4 and incident HF in Central and Eastern European populations remains limited. This evidence gap is particularly relevant given the considerable regional burden of T2DM and cardiovascular disease (11).

Serial FIB-4 may provide information about longitudinal hepatic and metabolic risk, but its clinical value should be distinguished from that of established cardiac biomarkers. The American Diabetes Association’s Standards of Care recommend considering BNP or NT-proBNP testing to screen for asymptomatic stage B heart failure in adults with diabetes (12). In addition, studies in people with type 2 diabetes have evaluated high-sensitivity cardiac troponin T alongside NT-proBNP for heart-failure risk assessment (13). FIB-4 is not a cardiac-specific biomarker and cannot replace natriuretic peptides, cardiac troponins, or cardiac imaging. In a general-population UK Biobank cohort, baseline FIB-4 was associated with incident heart-failure hospitalization or death (14). Longitudinal FIB-4 change has also been studied in people with obesity and/or type 2 diabetes, but this does not establish the predictive value of serial FIB-4 in an unselected general population (15). In the present cohort, NT-proBNP was available for only 54 of 8,221 participants. Troponin-I and troponin-T were stored as separate source variables and were not harmonized or included in the prediction models. Therefore, this study does not establish whether serial FIB-4 adds predictive value beyond NT-proBNP or high-sensitivity cardiac troponin. Any future role for serial FIB-4 would be complementary and requires direct evaluation against established cardiac biomarkers.

Therefore, this study investigated the associations of within-person FIB-4 variability and prespecified longitudinal FIB-4 patterns with incident HF among patients with T2DM treated at a tertiary clinical center in Hungary. We applied a 24-month landmark design comprising an 18-month FIB-4 assessment window and a subsequent 6-month lag period before the start of HF follow-up. Chronological age was controlled separately despite its inclusion in the FIB-4 calculation, and chronological temporal validation was used to assess the incremental discriminative contribution of serial FIB-4 features. We hypothesized that greater FIB-4 variability, persistent-high FIB-4, and worsening to a high FIB-4 category would be associated with a higher subsequent HF hazard.

## Materials and methods

### Study design and data source

This retrospective longitudinal cohort study was conducted as a secondary reanalysis of routinely collected, de-identified clinical data from the University of Debrecen Clinical Center, Hungary. No new patient recruitment, clinical examination, or laboratory sampling was performed for the present study. The source data originated from the institution’s electronic clinical records and included patient demographics, diagnosis codes, medication records, and laboratory measurements generated during routine care.

For analytical purposes, the source records had previously been harmonized into a longitudinal patient-month database, with one record per patient for each represented calendar month. The present reanalysis was performed on this consolidated monthly database using Python and PySpark in the Azure Databricks environment. The analysis was restricted to records between January 1, 2000, and December 31, 2025. The source table contained 27,887,496 patient-month records from 89,383 unique individuals. All cohort definitions, temporal eligibility criteria, FIB-4 calculations, outcome ascertainment, statistical models, validation procedures, and publication tables and figures were generated specifically for the present analysis.

T2DM was identified using the International Classification of Diseases, Tenth Revision (ICD-10) code E11. At least 12 months of recorded clinical history were required before an eligible index month; therefore, records from 2000 served as the earliest possible observation and washout period. This requirement reduced the likelihood that a diagnosis first appearing immediately after entry into the database would be incorrectly classified as an incident event.

### Cohort construction and landmark design

Patients were eligible for the source T2DM–HF cohort if they were aged at least 18 years, had an E11 diagnosis, were classified as having active T2DM in the index month, and had a valid same-month measurement of AST, ALT, and platelet count from which FIB-4 could be calculated. All three laboratory values were required to be positive and non-missing. The patient-specific index month was defined as the first month satisfying these criteria after at least 12 months of recorded clinical history.

The initial HF source cohort included 40,732 eligible patients. A 24-month landmark design was then applied. FIB-4 measurements from the index month (month 0) through month 18, inclusive, constituted the exposure-assessment window. This was followed by a 6-month exposure-free interval extending from the end of month 18 to the landmark at month 24; HF follow-up included only events occurring after the landmark. Patients were required to remain under observation beyond the landmark month and to be free of recorded HF up to and including that month. These criteria produced a landmark-eligible cohort of 26,141 patients.

Assessment of serial FIB-4 required at least two valid FIB-4 measurements during the 18-month exposure window. The first and last eligible measurements had to be separated by at least 6 months, and at least one measurement had to be available between months 12 and 18. Measurements recorded after month 18 were not used to define the serial exposure. After applying these serial-measurement criteria, the final analytical cohort consisted of 8,221 unique patients. Each patient contributed one record to the landmark survival analysis, thereby maintaining independence at the patient level.

### Outcome definition and follow-up

The primary outcome was incident HF, defined as the first recorded occurrence of ICD-10 code I50. A diagnosis recorded in or before the landmark month was considered prevalent HF, and the patient was excluded from the landmark cohort. An event was considered incident only when the first I50-coded month occurred strictly after the 24-month landmark and on or before the patient-specific censoring month.

As a sensitivity analysis, we applied a more restrictive coded-HF definition requiring a second distinct post-landmark month with an I50 code 7 to 365 days after the first; the event date was assigned to the first code in the pair. This analysis remained based on diagnostic codes and should not be interpreted as echocardiographic or clinically adjudicated HF. Because the unpenalized models did not converge under this endpoint definition, both models used a ridge penalty of 0.0001; confidence intervals and p-values were based on the approximate inverse penalized information matrix rather than robust sandwich variance estimates (**Supplementary Table S8**).

Follow-up began at the 24-month landmark. Patients were followed until the first recorded HF event or censoring, whichever occurred first. The censoring month was defined as the earlier of the last month with recorded clinical activity and December 2025. Clinical activity included any recorded diagnosis, medication, laboratory measurement, or other documented healthcare data. Because death dates were unavailable, death could not be incorporated as a competing event.

### FIB-4 calculation and serial exposure definitions

FIB-4 was calculated for every eligible patient-month containing simultaneous valid measurements of AST, ALT, and platelet count according to the formula described by Sterling et al. (8):


$$FIB-4=\frac{\text{Age }(\text{years})\;\times \text{AST }(\text{U}/\text{L})}{Platelet\;count\;(10^{9}/\text{ L})\;\times \sqrt{ALT(U/L)}}$$

Age at each measurement was obtained from the recorded age variable or calculated from year of birth and calendar year when necessary. Automated validation compared the stored FIB-4 values with values recalculated directly from the component variables.

The primary serial exposure was within-person FIB-4 variability, defined as the sample standard deviation of log_2_-transformed FIB-4 values recorded during the 18-month assessment window. Log_2_ transformation was used to reduce right skewness and express variability on a relative rather than an absolute scale. The number of FIB-4 measurements and the time span between the first and last measurements were also recorded.

For the categorical analysis, an FIB-4 value of 2.67 was used as the prespecified high-risk threshold (16). This threshold was not selected or optimized using HF outcomes. Patients were assigned to four mutually exclusive longitudinal patterns according to their first and last measurements within the exposure window:

- Never high: first and last FIB-4 values were both <2.67.
- Improving from high: the first FIB-4 value was ≥2.67, and the last value was <2.67.
- Worsening to high: the first FIB-4 value was <2.67, and the last value was ≥2.67.
- Persistent high: the first and last FIB-4 values were both ≥2.67.

The never-high pattern served as the reference category. All measurements used to calculate variability and longitudinal patterns preceded the 6-month lag and the start of HF follow-up.

### Clinical covariates

Demographic covariates included age and sex. Age at index was included separately in all primary adjusted models and expressed per 10-year increase. Although age is a component of FIB-4, separate adjustment was retained because chronological age is an independent determinant of HF.

The duration of recorded diabetes was calculated as the interval between the first recorded E11 diagnosis and the patient-specific index month. This variable therefore represented time since the first recorded T2DM code rather than the true clinical duration of diabetes.

Comorbidities recorded on or before the index month included hypertension (I10), atrial fibrillation (I48), chronic kidney disease (N18), and obesity (E66). For these chronic conditions, the entire available diagnostic history up to the index month was considered. Medication exposure was defined using prescriptions recorded during the 365 days ending in the index month and included statins (Anatomical Therapeutic Chemical [ATC] codes C10AA or C10B), renin–angiotensin–aldosterone system inhibitors (C09), antiplatelet agents (B01AC), sodium–glucose cotransporter-2 inhibitors (A10BK), and glucagon-like peptide-1 receptor agonists (A10BJ). The absence of a recorded diagnosis or medication code was interpreted as no documented condition or exposure in the available data, rather than proof of clinical absence.

For sensitivity analyses, pre-index cardiovascular history was derived from all available diagnoses recorded up to and including the index month. It comprised previous myocardial infarction (I21–I22), other ischemic heart disease (I20 and I23–I25), stroke (I60–I64), and peripheral arterial disease (I70 or I73). The models additionally included the logarithmically transformed number of FIB-4 measurements to account for differences in measurement intensity. Landmark calendar time was incorporated to account for temporal changes in diagnostic practices, data availability, and T2DM treatment during the extended study period.

Cardiac biomarkers were not included in the primary or expanded Cox models. NT-proBNP was available for 54 of 8,221 participants (0.7%). Troponin-I was available for 34 participants (0.4%; 6 subsequent HF events) and troponin-T for 431 participants (5.2%; 119 subsequent HF events) during the 365-day period before index. The monthly source table contained 35 non-missing troponin-I and 482 non-missing troponin-T patient-month records; these counts do not represent individual laboratory tests. Troponin-I and troponin-T were retained as separate variables, and assay metadata identifying high-sensitivity measurements were unavailable. No troponin-adjusted model or direct incremental-prediction comparison with cardiac biomarkers was performed.

### Descriptive and time-to-event analyses

Continuous variables were summarized as medians and interquartile ranges, and categorical variables as numbers and percentages. Characteristics were presented for the overall cohort and according to subsequent HF status. Between-group differences were quantified using standardized mean differences. Serial FIB-4 patterns were summarized using patient numbers, HF events, accumulated person-years, crude event proportions, and incidence rates per 1,000 person-years.

Unadjusted HF-free survival was estimated using the Kaplan–Meier method. For graphical presentation, the probability of recorded HF was displayed as the Kaplan–Meier failure estimate, $1-\hat{S}(t)$, with corresponding numbers at risk. Because death was unavailable, these estimates were not interpreted as competing-risk cumulative incidence functions.

### Cox proportional hazards models

Associations with incident HF were estimated using Cox proportional hazards regression with follow-up time measured from the 24-month landmark. Separate multivariable models were fitted for within-person FIB-4 variability and the four-category longitudinal FIB-4 pattern.

The variability model estimated the HF hazard associated with a one-unit increase in the within-person standard deviation of log_2_-transformed FIB-4. In the pattern model, improving-from-high, worsening-to-high, and persistent-high patterns were compared with the never-high reference group.

The primary models adjusted for age per 10 years, duration of recorded T2DM, sex, atrial fibrillation, chronic kidney disease, obesity, statin use, renin–angiotensin–aldosterone system inhibitor use, antiplatelet use, and log_2_-transformed FIB-4 measurement count. Sodium–glucose cotransporter-2 inhibitor and glucagon-like peptide-1 receptor agonist use was not included in the primary models because recorded exposure was extremely sparse; their inclusion was evaluated in a medication-related sensitivity analysis. Hypertension, baseline FIB-4, and landmark calendar period were handled by stratification.

Baseline log_2_-transformed FIB-4 and landmark calendar year were divided into outcome-independent tertiles. Joint strata were constructed from baseline FIB-4 tertile, calendar-period tertile, and hypertension status. To avoid sparse strata, baseline FIB-4 tertiles were combined within the most recent calendar-period stratum among patients without recorded hypertension. No patients were excluded, and the resulting 16 joint strata each contained at least five HF events and five non-events. The stratified models allowed separate baseline hazard functions across strata while retaining explicit adjustment for chronological age.

A small penalization parameter of 10^−4^ was applied to improve numerical stability, and robust variance estimates were used. Associations were reported as hazard ratios (HRs) with 95% confidence intervals (CIs). The proportional-hazards assumption was evaluated using tests based on scaled Schoenfeld residuals with rank-transformed event time. The proportional-hazards results for the principal FIB-4 terms were reported separately.

### Chronological temporal validation

Temporal validation used an earlier training cohort and a later test cohort defined by the prespecified calendar-time split. We compared a baseline model containing clinical covariates, landmark calendar time, and baseline FIB-4 with an extended model that additionally included the serial FIB-4 terms. Discrimination was assessed using Harrell’s C-index. We also assessed calibration and overall prediction error in the test cohort using calibration-in-the-large, the calibration slope, observed-to-expected ratios, and Brier scores at 1 and 2 years. The change in C-index was estimated using bootstrap resampling. Baseline characteristics of the training and test cohorts are reported in **Supplementary Table S6**; performance and calibration metrics are reported in **Supplementary Table S5**, with calibration plots in **Supplementary Figure S1**.

### Sensitivity analysis

Two sensitivity analyses evaluated potential confounding by preexisting cardiovascular disease. First, the full-cohort variability and pattern models were additionally adjusted for previous myocardial infarction, other ischemic heart disease, stroke, and peripheral arterial disease. Second, the models were repeated after excluding all patients with any of these diagnoses recorded up to and including the index month. Both analyses used the same landmark design, exposure definitions, core covariates, stratification structure, penalization, robust variance estimation, and proportional-hazards diagnostics as the primary analyses.

Medication-related sensitivity analyses evaluated the robustness of the findings to alternative medication-covariate specifications. First, models were fitted with all five prespecified medication classes, adding sodium–glucose cotransporter-2 inhibitor and glucagon-like peptide-1 receptor agonist use to the three medication classes included in the primary models. Second, the models were repeated without any medication covariates. Medication exposure was also independently reconstructed using ATC-prefix matching, which reproduced the original exposure classifications.

Additional sensitivity analyses addressed the availability of metabolic and lifestyle-related covariates. HbA1c was available for 4,214 of 8,221 participants (51.3%). Missing HbA1c values were imputed using chained-equation multiple imputation to create 20 imputed datasets. Expanded Cox models were fitted separately in each imputed dataset, and coefficient estimates and within-imputation variances were pooled using Rubin’s rules. The within-imputation variances were model-based; robust sandwich variance estimates were not used for these sensitivity models. Continuous BMI, systolic and diastolic blood pressure, and measured smoking and alcohol consumption were unavailable and therefore could not be included. Separate models included recorded-diagnosis proxies for MASLD, tobacco use, and alcohol-related diagnoses; these proxies do not represent measured lifestyle exposures. Results are presented in **Supplementary Table S7**.

### Data integrity and reproducibility

The analytical workflow incorporated automated checks of patient-level uniqueness, temporal ordering, FIB-4 calculation, exposure classification, event coding, follow-up time, model strata, proportional-hazards diagnostics, temporal validation, and agreement between analytical and publication outputs. All 78 predefined validation checks were completed without warnings.

All data management and statistical analyses were performed using Python within Azure Databricks, primarily with PySpark, pandas, NumPy, lifelines, and scikit-learn. Two-sided p-values <0.05 were considered statistically significant.

### Ethics statement

The study was conducted in accordance with the Declaration of Helsinki and was approved by the Scientific and Research Ethics Committee of the Health Science Council (BM/11356-1/2025; 8 May 2025). The requirement for individual informed consent was waived because the study involved secondary analysis of deidentified data.

## Results

### Cohort selection and participant characteristics

The source monthly database contained 27,887,496 patient-month records from 89,383 unique individuals. A total of 357,460 valid monthly FIB-4 measurements were available from 55,431 patients. After applying the age, T2DM, observation-history, laboratory-availability, and outcome eligibility criteria, the HF source cohort included 40,732 patients. Of these, 26,141 remained under observation beyond the 24-month landmark and were free of recorded HF up to and including that month. Application of the serial-measurement requirements resulted in a final analytical cohort of 8,221 patients.

During 50,640.1 person-years of post-landmark follow-up, 1,811 patients developed incident HF and 6,410 remained free of recorded HF, corresponding to an overall event proportion of 22.0% and a crude incidence rate of 35.8 events per 1,000 person-years (**Figure 1**).

**Figure 1:**
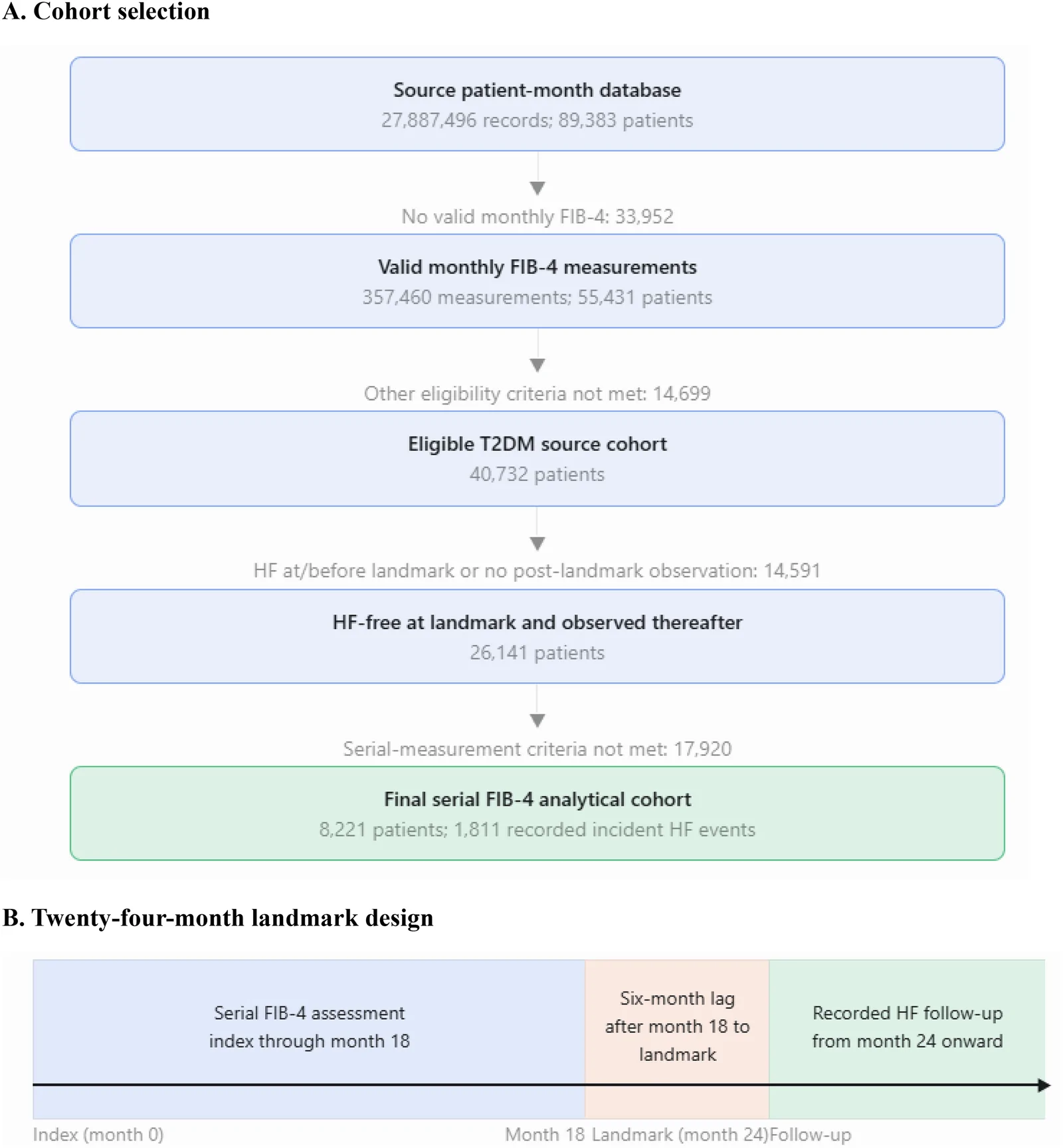
Study cohort selection and 24-month landmark design. **(A)** Cohort selection **(B)** 24-month landmark design **(A)** Sequential construction of the analytical cohort from the longitudinal patient-month database. Of 89,383 patients represented in the source database, 55,431 had at least one valid monthly FIB-4 measurement, 40,732 met the initial T2DM cohort criteria, and 26,141 remained under observation and were free of recorded heart failure at the 24-month landmark. Application of the prespecified serial-measurement requirements resulted in a final analytical cohort of 8,221 patients, among whom 1,811 developed incident heart failure during post-landmark follow-up. **(B)** Serial FIB-4 measurements obtained from the patient-specific index month through month 18 constituted the exposure-assessment window. This was followed by a 6-month exposure-free lag, with follow-up for recorded incident heart failure beginning strictly after the month-24 landmark and continuing until the first recorded HF event or censoring.

The median age at index was 61 years (interquartile range [IQR], 52–69 years), and 4,428 participants (53.9%) were women (**Table 1**). Patients who developed HF were older than those without incident HF (median, 64 [IQR, 56–72] versus 60 [IQR, 51–68] years; standardized mean difference [SMD], 0.417). Hypertension was recorded in 82.4% of patients with incident HF and 73.5% of those without HF (SMD, 0.215). Atrial fibrillation was present in 9.0% and 4.4%, respectively (SMD, 0.186), whereas chronic kidney disease was present in 19.7% and 13.5%, respectively (SMD, 0.166).

**Table 1:** Characteristics of the analytical cohort according to incident recorded heart failure status.

Characteristic	Overall (N = 8,221)	No incident HF (N = 6,410)	Incident HF (N = 1,811)	SMD
Age, years	61.0 [52.0, 69.0]	60.0 [51.0, 68.0]	64.0 [56.0, 72.0]	0.417
Recorded diabetes duration, years	0.2 [0.0, 1.5]	0.2 [0.0, 1.5]	0.2 [0.0, 1.5]	−0.063
Baseline FIB-4	1.12 [0.78, 1.59]	1.08 [0.75, 1.55]	1.24 [0.90, 1.71]	0.138
Last FIB-4 during exposure window	1.16 [0.81, 1.62]	1.13 [0.79, 1.58]	1.27 [0.90, 1.75]	0.162
Number of FIB-4 measurements	4 [3, 5]	4 [3, 5]	4 [2, 5]	−0.016
FIB-4 variability, SD of log_2_(FIB-4)	0.238 [0.145, 0.365]	0.237 [0.144, 0.361]	0.241 [0.150, 0.380]	0.039
FIB-4 slope, log_2_ units/year	0.040 [−0.166, 0.257]	0.046 [−0.161, 0.260]	0.021 [−0.179, 0.242]	−0.029
Female sex	4,428 (53.9%)	3,504 (54.7%)	924 (51.0%)	−0.073
Hypertension	6,204 (75.5%)	4,712 (73.5%)	1,492 (82.4%)	0.215
Atrial fibrillation	443 (5.4%)	280 (4.4%)	163 (9.0%)	0.186
Chronic kidney disease	1,221 (14.9%)	865 (13.5%)	356 (19.7%)	0.166
Obesity	1,270 (15.4%)	1,000 (15.6%)	270 (14.9%)	−0.019
Statin use	348 (4.2%)	271 (4.2%)	77 (4.3%)	0.001
RAAS inhibitor use	586 (7.1%)	449 (7.0%)	137 (7.6%)	0.022
Antiplatelet use	454 (5.5%)	326 (5.1%)	128 (7.1%)	0.083
SGLT2 inhibitor use	7 (0.1%)	7 (0.1%)	0 (0.0%)	−0.047
GLP-1 receptor agonist use	97 (1.2%)	96 (1.5%)	1 (0.1%)	−0.165

Values are median [interquartile range] or n (%). SMDs compare patients with versus without incident recorded HF; positive values indicate a higher mean or prevalence in the HF group. Medication exposure was based on recorded prescriptions during the 365 days ending in the index month. Abbreviations: FIB-4, Fibrosis-4 index; GLP-1, glucagon-like peptide-1; HF, heart failure; RAAS, renin–angiotensin–aldosterone system; SGLT2, sodium–glucose cotransporter-2; SMD, standardized mean difference.

The median baseline FIB-4 was 1.12 (IQR, 0.78–1.59) in the overall cohort. Baseline FIB-4 was higher among patients who subsequently developed HF than among those who did not (1.24 [IQR, 0.90–1.71] versus 1.08 [IQR, 0.75–1.55]; SMD, 0.138). The corresponding last FIB-4 values within the exposure window were 1.27 (IQR, 0.90–1.75) and 1.13 (IQR, 0.79–1.58) (SMD, 0.162). Patients contributed a median of four FIB-4 measurements (IQR, 3–5). The median within-person standard deviation of log_2_-transformed FIB-4 was 0.238 (IQR, 0.145–0.365) and was similar in the crude HF and non-HF groups (0.241 versus 0.237; SMD, 0.039).

Recorded medication exposure was infrequent. Statin use was recorded in 348 patients (4.2%), renin–angiotensin–aldosterone system inhibitor use in 586 (7.1%), antiplatelet use in 454 (5.5%), sodium–glucose cotransporter-2 inhibitor use in 7 (0.1%), and glucagon-like peptide-1 receptor agonist use in 97 (1.2%).

### Serial FIB-4 patterns and heart failure occurrence

Most patients were classified as never high (n=7,401; 90.0%), whereas 252 (3.1%) improved from high, 277 (3.4%) worsened to high, and 291 (3.5%) had persistent-high FIB-4 (**Table 2** and **Supplementary Table S1**). The median baseline and last FIB-4 values were 1.05 and 1.09 in the never-high group, 3.23 and 1.81 in the improving-from-high group, 2.01 and 3.22 in the worsening-to-high group, and 4.26 and 4.05 in the persistent-high group. The interval-specific distributions of FIB-4 during the 18-month exposure window are shown in **Figure 2**.

**Table 2:** Serial FIB-4 patterns and crude occurrence of incident recorded heart failure.

Serial FIB-4 pattern	Patients, n	HF events, n (%)	Person-years	Incidence rate per 1,000 person-years	Median follow-up, years	Median baseline FIB-4	Median last FIB-4	Median log_2_ FIB-4 slope/year
Never high	7,401	1,603 (21.7%)	47,274.5	33.9	5.08	1.05	1.09	0.038
Improving from high	252	47 (18.7%)	1,116.3	42.1	2.50	3.23	1.81	−0.588
Worsening to high	277	72 (26.0%)	1,177.2	61.2	2.75	2.01	3.22	0.600
Persistent high	291	89 (30.6%)	1,072.1	83.0	2.25	4.26	4.05	0.054

Serial patterns were defined from the first and last FIB-4 measurements within the 18-month exposure window using the prespecified threshold of 2.67. Follow-up for recorded HF began at the 24-month landmark. Incidence rates are reported per 1,000 person-years. Abbreviations: FIB-4, Fibrosis-4 index; HF, heart failure.

**Figure 2:**
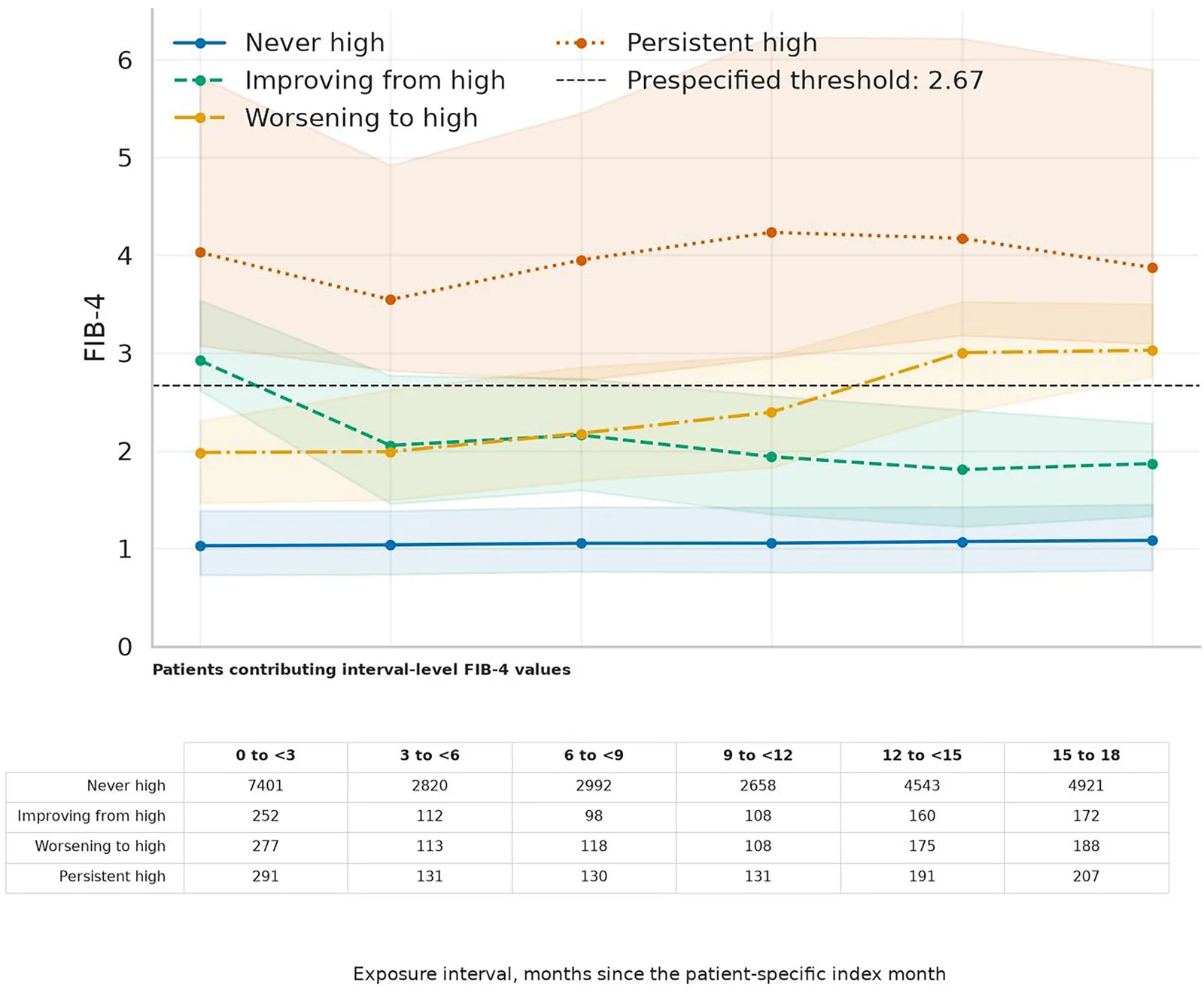
Serial FIB-4 distributions during the 18-month exposure-assessment window. FIB-4 measurements are summarized across six consecutive 3-month intervals according to longitudinal FIB-4 pattern. When a patient had multiple measurements within an interval, the patient-specific median was calculated first to prevent frequently tested patients from disproportionately influencing the estimates. Lines represent the median of these patient-level values, and shaded areas represent the interquartile range. The horizontal dashed line indicates the prespecified FIB-4 threshold of 2.67.

Among patients with a never-high pattern, 1,603 HF events occurred during 47,274.5 person-years, corresponding to a crude incidence rate of 33.9 per 1,000 person-years. The improving-from-high group experienced 47 events during 1,116.3 person-years, corresponding to 42.1 events per 1,000 person-years. In the worsening-to-high group, 72 events occurred during 1,177.2 person-years, corresponding to 61.2 events per 1,000 person-years. The persistent-high group experienced 89 events during 1,072.1 person-years, corresponding to 83.0 events per 1,000 person-years.

The proportions experiencing HF were 21.7% in the never-high group, 18.7% in the improving-from-high group, 26.0% in the worsening-to-high group, and 30.6% in the persistent-high group. The Kaplan–Meier failure estimates showed separation among the serial FIB-4 patterns during post-landmark follow-up, with the highest estimated HF probability observed in the persistent-high group (**Figure 3**).

**Figure 3:**
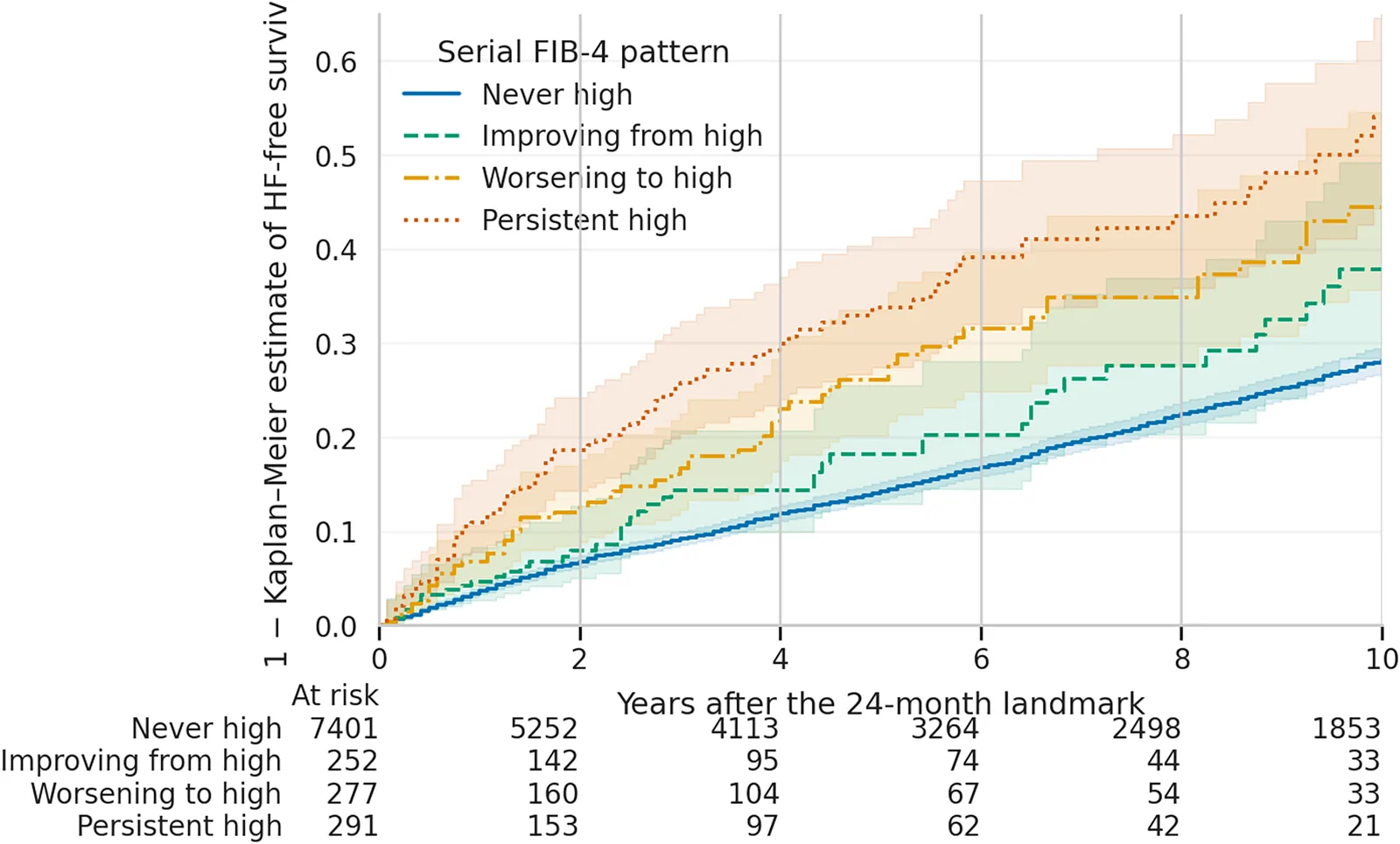
Kaplan–Meier failure estimates for incident heart failure according to serial FIB-4 pattern. Unadjusted Kaplan–Meier failure estimates are presented for the never-high, improving-from-high, worsening-to-high, and persistent-high FIB-4 patterns. Shaded areas represent 95% confidence intervals, and numbers at risk are displayed below the graph. Time was measured from the 24-month landmark, and graphical presentation was restricted to the first 10 years of post-landmark follow-up. Because death data were unavailable, these estimates should not be interpreted as competing-risk cumulative incidence functions.

### Multivariable associations with incident heart failure

In the primary multivariable model stratified by baseline FIB-4, landmark calendar period, and hypertension status, greater within-person FIB-4 variability was associated with a higher hazard of incident HF (HR per one-unit increase in the standard deviation of log_2_-transformed FIB-4, 1.73; 95% CI, 1.43–2.09; p<0.001; **Table 3** and **Figure 4**).

**Table 3:** Associations of serial FIB-4 features with incident recorded heart failure.

Specification	Exposure	HR (95% CI)	P value
Primary stratified model	FIB-4 variability (per 1-unit higher SD of log_2_ FIB-4)	1.73 (1.43–2.09)	<0.001
Secondary conventionally adjusted model	FIB-4 variability (per 1-unit higher SD of log_2_ FIB-4)	1.67 (1.38–2.02)	<0.001
Primary stratified model	Persistent high vs. never high	1.73 (1.35–2.20)	<0.001
Secondary conventionally adjusted model	Persistent high vs. never high	1.69 (1.28–2.24)	<0.001
Primary stratified model	Worsening to high vs. never high	1.28 (1.01–1.62)	0.043
Secondary conventionally adjusted model	Worsening to high vs never high	1.27 (1.00–1.61)	0.053
Primary stratified model	Improving from high vs. never high	0.95 (0.70–1.28)	0.730
Secondary conventionally adjusted model	Improving from high vs. never high	0.93 (0.67–1.28)	0.652

The primary models were stratified by baseline FIB-4 tertile, landmark calendar period, and hypertension status and separately adjusted for chronological age and the clinical covariates specified in the Methods. Secondary models used conventional covariate adjustment. The never-high pattern was the reference category. Abbreviations: CI, confidence interval; FIB-4, Fibrosis-4 index; HF, heart failure; HR, hazard ratio.

**Figure 4:**
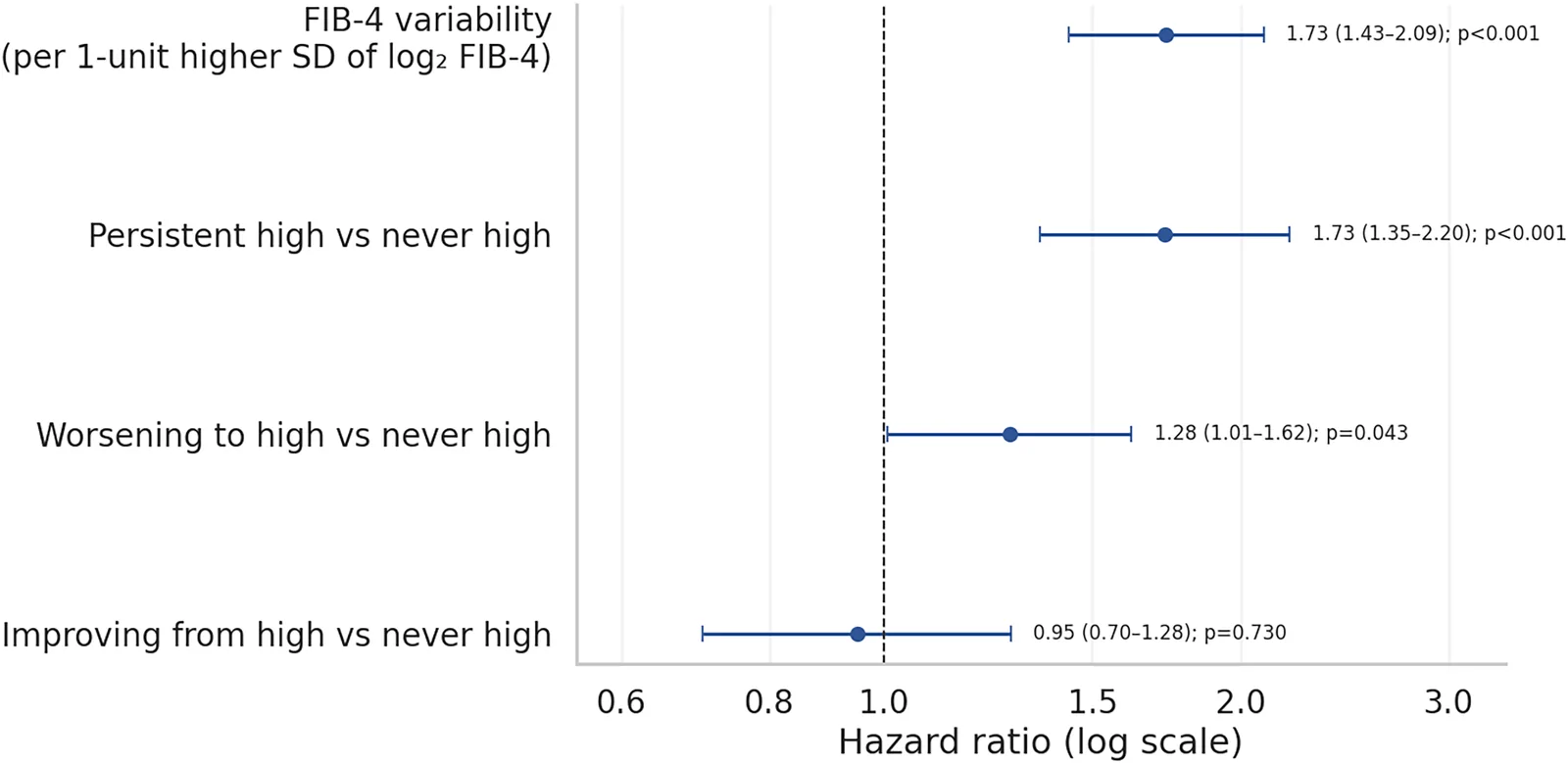
Adjusted associations of FIB-4 variability and longitudinal patterns with incident heart failure. Points represent adjusted hazard ratios, and horizontal lines represent 95% confidence intervals from the final stratified Cox proportional hazards models. The estimate for FIB-4 variability represents a one-unit increase in the within-person standard deviation of log_2_-transformed FIB-4. Longitudinal-pattern estimates compare persistent-high, worsening-to-high, and improving-from-high FIB-4 with the never-high reference pattern. Models were adjusted for the clinical covariates specified in the Methods and stratified by baseline FIB-4, landmark calendar period, and hypertension status. Hazard ratios are displayed on a logarithmic scale.

In the longitudinal-pattern model, persistent-high FIB-4 was associated with a higher HF hazard than the never-high pattern (HR, 1.73; 95% CI, 1.35–2.20; p<0.001). Worsening to high was also associated with an increased hazard (HR, 1.28; 95% CI, 1.01–1.62; p=0.043). In contrast, improving from high was not associated with HF relative to the never-high group (HR, 0.95; 95% CI, 0.70–1.28; p=0.730).

Secondary non-stratified models produced similar estimates for FIB-4 variability (HR, 1.67; 95% CI, 1.38–2.02; p<0.001) and persistent-high FIB-4 (HR, 1.69; 95% CI, 1.28–2.24; p<0.001). Worsening to high showed a borderline association (HR, 1.27; 95% CI, 1.00–1.61; p=0.053), whereas improving from high remained unassociated with HF (HR, 0.93; 95% CI, 0.67–1.28; p=0.652).

The principal findings were also materially unchanged after ATC-prefix-based reconstruction of medication exposure, inclusion of all five medication classes, and omission of all medication covariates.

### Proportional-hazards diagnostics and temporal validation

All principal FIB-4 terms satisfied the proportional-hazards diagnostic. The corresponding p-values were 0.667 for FIB-4 variability, 0.778 for worsening to high, 0.528 for improving from high, and 0.775 for persistent-high FIB-4 (**Supplementary Table S2**). The final stratification procedure produced 16 joint strata, all of which contained at least five HF events and five non-events (**Supplementary Table S3**).

The training cohort included 6,604 participants and 1,734 HF events, whereas the temporally later test cohort included 1,617 participants and 77 events. In the test cohort, Harrell’s C-index was 0.7081 for the baseline model and 0.7150 for the extended model; the difference was 0.0069 (95% CI, −0.0043 to 0.0190). The temporal-validation discrimination results are summarized in **Table 4**. Brier scores were 0.0349 and 0.0390 for the baseline model and 0.0349 and 0.0389 for the extended model at 1 and 2 years, respectively. Calibration-in-the-large and calibration slopes are reported in **Supplementary Table S5**. The cohorts differed in calendar time and follow-up duration, and the test cohort had relatively few HF events; therefore, these temporal-validation results should be interpreted cautiously (**Supplementary Table S6** and **Supplementary Figure S1**).

**Table 4:** Chronological temporal validation of serial FIB-4 features.

Model	Split date	Training cohort, n/events	Test cohort, n/events	Harrell C-index	ΔC-index vs. baseline	Bootstrap 95% CI for ΔC-index	Bootstrap repetitions
Baseline clinical model plus baseline FIB-4	2023-08-01	6,604/1,734	1,617/77	0.7081	Reference	Reference	—
Baseline model plus serial FIB-4 features	2023-08-01	6,604/1,734	1,617/77	0.7150	0.0069	−0.0043 to 0.0190	300

Patients were divided chronologically into an earlier 80% training cohort and a later 20% test cohort. Models were fitted in the training cohort and evaluated without refitting in the test cohort. The confidence interval for the change in C-index was obtained from 300 bootstrap resamples of the test cohort. Abbreviations: CI, confidence interval; FIB-4, Fibrosis-4 index.

### Sensitivity analyses

In the full cohort, additional adjustment for pre-index myocardial infarction, other ischemic heart disease, stroke, and peripheral arterial disease did not materially alter the principal estimates (**Supplementary Table S4**). FIB-4 variability remained associated with incident HF (HR, 1.75; 95% CI, 1.45–2.12; p<0.001), as did persistent-high FIB-4 (HR, 1.73; 95% CI, 1.35–2.22; p<0.001) and worsening to high (HR, 1.27; 95% CI, 1.00–1.61; p=0.047). Improving from high remained unassociated with HF (HR, 0.94; 95% CI, 0.69–1.27; p=0.673).

After excluding 4,459 patients with documented pre-index cardiovascular disease, the restricted cohort comprised 3,762 patients, including 605 incident HF events. In this cohort, FIB-4 variability (HR, 1.90; 95% CI, 1.37–2.64; p<0.001) and persistent-high FIB-4 (HR, 2.34; 95% CI, 1.59–3.43; p<0.001) remained associated with HF. Worsening to high (HR, 1.24; 95% CI, 0.77–2.02; p=0.376) and improving from high (HR, 0.83; 95% CI, 0.46–1.51; p=0.542) were not associated with HF in the restricted cohort.

In the HbA1c-adjusted multiple-imputation sensitivity analysis, the association remained for FIB-4 variability (HR, 1.61; 95% CI, 1.33–1.95; p<0.001) and persistent-high FIB-4 (HR, 1.71; 95% CI, 1.36–2.15; p<0.001). Worsening to high was not statistically significant (HR, 1.27; 95% CI, 0.99–1.62; p=0.056), and improving from high remained unassociated with HF (HR, 0.92; 95% CI, 0.68–1.24; p=0.571). Estimates were similar in models additionally including recorded-diagnosis proxies for MASLD, tobacco use, and alcohol-related diagnoses: HR 1.57 (95% CI, 1.30–1.91; p<0.001) for variability; 1.68 (1.34–2.11; p<0.001) for persistent-high FIB-4; 1.25 (0.97–1.59; p=0.079) for worsening to high; and 0.91 (0.67–1.23; p=0.531) for improving from high (**Supplementary Table S7**).

Under the more restrictive definition requiring two post-landmark I50-coded months, 833 HF events were identified. FIB-4 variability remained associated with HF (HR, 1.37; 95% CI, 1.01–1.84; p=0.040). The pattern estimates were less precise and were not statistically significant: persistent-high FIB-4, HR 0.80 (95% CI, 0.50–1.26; p=0.329); worsening to high, HR 1.18 (0.82–1.70; p=0.361); and improving from high, HR 0.89 (0.57–1.39; p=0.607) (**Supplementary Table S8**).

## Discussion

In this longitudinal real-world cohort of 8,221 patients with T2DM who were free of recorded HF at landmark entry, greater within-person FIB-4 variability and persistent-high FIB-4 were associated with a higher subsequent hazard of incident HF. These associations remained present after explicit adjustment for chronological age and other clinical characteristics and after stratification by baseline FIB-4, calendar period, and hypertension status. Worsening from below to above the prespecified FIB-4 threshold was associated with a more modest increase in HF hazard, whereas improvement from a high FIB-4 category was not associated with subsequent HF. The associations of FIB-4 variability and persistent-high FIB-4 also remained present after adjustment for pre-index cardiovascular disease and after restricting the analysis to patients without documented pre-index cardiovascular disease. Although serial FIB-4 features were associated with HF, their addition to a baseline clinical model produced only a small and statistically uncertain increase in discrimination during chronological temporal validation.

Previous studies have demonstrated associations between elevated FIB-4 and cardiovascular morbidity and mortality in patients with T2DM (9, 10). FIB-4 has also been linked to cardiovascular outcomes in populations with MASLD and other forms of metabolic liver dysfunction (17, 18). It has been proposed as a first-line fibrosis-stratification tool in metabolic liver disease (16), whereas a high FIB-4 burden is frequently observed in T2DM (19). FIB-4 and other non-invasive liver fibrosis scores have also shown prognostic associations with mortality in patients with acute myocardial infarction and with mortality and liver-related outcomes in NAFLD cohorts (20, 21). However, most previous studies relied on a single FIB-4 measurement or conventional baseline category. The present study extends this evidence by evaluating within-person variability and longitudinal patterns during a defined 18-month assessment window followed by a 6-month exposure-free lag. The landmark structure ensured that all FIB-4 measurements used to define the exposures preceded the HF events included in the analysis.

The association between FIB-4 and HF may reflect several overlapping processes. MASLD and hepatic fibrosis share metabolic and inflammatory features with HF, including insulin resistance, oxidative stress, endothelial dysfunction, and chronic low-grade inflammation (7). Renal dysfunction may further contribute to HF risk within this multisystem cardiometabolic framework (22). Conversely, early cardiac dysfunction or venous congestion may alter aminotransferase levels and platelet-related parameters before HF is clinically recognized. FIB-4 should therefore not be interpreted exclusively as a direct measure of hepatic fibrosis in this setting. Instead, it may represent a composite indicator of age, hepatic or systemic injury, hematological changes, and overall multisystem cardiometabolic burden.

Greater within-person FIB-4 variability was consistently associated with incident HF. Such variability could reflect fluctuations in metabolic or inflammatory activity, hepatic or renal status, intercurrent illness, medication exposure, or early hemodynamic instability. However, it may also be influenced by analytical variation and differences in the number and timing of laboratory measurements. We therefore required temporally separated measurements, adjusted for measurement frequency, and introduced a 6-month lag between exposure assessment and HF follow-up. These measures reduced, but could not eliminate, the influence of healthcare utilization, measurement error, or subclinical HF already developing during the exposure window.

Persistent-high FIB-4 was associated with an approximately 73% higher adjusted HF hazard than the never-high pattern. Persistent elevation may be less sensitive to an isolated abnormal laboratory result and may identify patients with a sustained burden of hepatic or systemic cardiometabolic dysfunction. Worsening to high was associated with a smaller increase in the primary model. This finding requires caution because the group contained only 277 patients and 72 HF events, its confidence interval was close to the null, and the association was not retained in the cohort without documented pre-index cardiovascular disease. It should therefore be considered secondary rather than definitive.

Improvement from high was not associated with a lower HF hazard. This does not establish that reducing FIB-4 has no clinical benefit. The improving group was small, with only 47 HF events, and may have included transient initial elevations, regression to the mean, treatment-related changes, or recovery from intercurrent illness. Because the reasons for FIB-4 changes were unavailable, the observed longitudinal patterns cannot be interpreted as treatment effects or causal disease trajectories.

The explicit handling of chronological age was an important methodological feature. Age is a direct component of FIB-4 and is independently associated with HF; failure to control for it could therefore attribute part of the age-related HF hazard to FIB-4. Age was retained as a separate covariate in every primary model, whereas baseline FIB-4 was handled through stratification. The principal FIB-4 terms satisfied the proportional-hazards diagnostic, and estimates from the stratified and conventionally adjusted models were similar. These findings reduce concern that the observed associations were explained solely by age or by one particular model specification, although residual confounding cannot be excluded.

The temporal-validation findings do not establish clinically meaningful incremental prediction from serial FIB-4: the increase in C-index was small, its confidence interval included zero, and Brier scores were nearly unchanged. Current diabetes guidance supports considering BNP or NT-proBNP to screen for asymptomatic stage B HF; serial FIB-4 should therefore not be viewed as a substitute for natriuretic peptides, cardiac troponins, or cardiac imaging. Its potential future role is complementary and remains unproven. If validated in independent cohorts, automated review of routinely available FIB-4 values might help identify patients with persistently high or increasingly variable values for whom clinicians could consider a broader hepatic and cardiovascular assessment. Such use would require evidence that serial FIB-4 adds information beyond established clinical predictors and cardiac biomarkers, followed by prospective evaluation of clinical impact. The present study does not support FIB-4-guided screening, diagnosis, or treatment. NT-proBNP was available for 54 of 8,221 participants, whereas troponin-I and troponin-T were available for 34 participants (6 subsequent HF events) and 431 participants (119 subsequent HF events), respectively; assay metadata identifying high-sensitivity measurements were unavailable, and no biomarker-adjusted prediction models were fitted, so the incremental predictive value of serial FIB-4 beyond these cardiac biomarkers remains unknown.

The generalizability of serial FIB-4 findings beyond people with type 2 diabetes is uncertain. A large UK Biobank study associated baseline FIB-4 with incident HF hospitalization or death in a general-population cohort, whereas longitudinal FIB-4-change evidence has primarily been reported in populations with obesity and/or type 2 diabetes (14, 15). These studies provide a rationale for evaluating serial FIB-4 in broader populations but do not establish that repeated measurements improve HF prediction in the general population. This question requires dedicated external validation.

## Strengths and limitations

The strengths of this study include its large real-world cohort, extended calendar period, patient-level longitudinal structure, repeated FIB-4 measurements, and strict temporal separation of exposure assessment from HF follow-up. The landmark design reduced the potential for reverse temporal classification, whereas the 6-month lag limited the possibility that laboratory changes recorded immediately before an HF diagnosis were treated as long-term predictors. Additional strengths included separate age control, adjustment for measurement frequency, robust variance estimation, proportional-hazards diagnostics, chronological temporal validation, and automated checks of cohort integrity and consistency between analytical and publication outputs. The consistency of the principal findings after adjustment for pre-index cardiovascular disease and in the restricted CVD-free cohort provided additional robustness.

Several limitations should be acknowledged. First, this was a retrospective analysis of routinely collected data from a single tertiary clinical center, which may limit generalizability. Eligibility required repeated and temporally separated FIB-4 measurements and continued observation through the 24-month landmark. The results therefore apply to patients who survived, remained under observation, and underwent sufficient laboratory monitoring to enter the landmark cohort. This may have introduced selection and healthcare-utilization bias.

Second, HF was identified from the first recorded ICD-10 I50 code and was not independently adjudicated. Information on symptoms, hospitalization, echocardiographic findings, left ventricular ejection fraction, natriuretic peptide concentrations, and HF phenotype was not consistently available. HF with preserved and reduced ejection fraction could therefore not be distinguished. Death dates were also unavailable, preventing competing-risk analysis, and censoring at the last recorded clinical activity may have been informative. The two-code sensitivity analysis provided a more restrictive coded endpoint, but the absence of clinical adjudication means that outcome misclassification remains possible. Cardiac biomarker comparisons were limited by sparse availability. NT-proBNP was available for 54 of 8,221 participants. During the index month and preceding 365 days, troponin-I was available for 34 participants (6 subsequent HF events) and troponin-T for 431 participants (119 subsequent HF events). These are participant counts based on non-missing monthly aggregated values, not counts of individual laboratory tests. Assay metadata identifying high-sensitivity measurements were unavailable. No biomarker-adjusted models or direct incremental-prediction comparisons were performed; therefore, this study cannot establish whether serial FIB-4 adds predictive value beyond NT-proBNP or high-sensitivity cardiac troponin.

Third, residual confounding may remain because continuous BMI, blood-pressure measurements, and measured smoking and alcohol consumption were unavailable. Although recorded obesity was included in the primary models, it may not fully capture adiposity. HbA1c was available for approximately half of the cohort and was addressed in a multiple-imputation sensitivity analysis. Recorded MASLD, tobacco-use, and alcohol-related diagnoses were examined as proxies, but these variables do not measure disease severity or lifestyle exposure and may be under-recorded. Preexisting cardiovascular disease was addressed through additional adjustment and cohort restriction, although diagnoses may have been incompletely ascertained. Other covariates were measured at or before index and were not modeled as time-varying exposures.

Fourth, medication exposure was likely incompletely captured. Recorded use of statins, renin–angiotensin–aldosterone system inhibitors, SGLT2 inhibitors, and GLP-1 receptor agonists was substantially lower than would be expected in a contemporary T2DM population. The absence of a medication code therefore cannot be interpreted as confirmed non-use. Changes in treatment during follow-up were also not modeled, despite the increasing use of therapies with cardiovascular, renal, and hepatic effects (23–25).

Fifth, FIB-4 is an indirect fibrosis index rather than a liver-specific biomarker. Its components may be influenced by acute illness, hematological conditions, medication use, alcohol intake, and non-hepatic sources of aminotransferase elevation. The threshold of 2.67 was not specifically validated as an HF threshold in this population. Classification based on the first and last measurements may have been affected by regression to the mean and did not capture every intermediate change, although the variability analysis incorporated all eligible measurements.

Finally, the temporally later test cohort included only 77 HF events and differed substantially from the training cohort in calendar time and follow-up duration, limiting the precision and transportability of the discrimination and calibration estimates; external validation in larger independent cohorts with more events is needed.

## Conclusion

In patients with T2DM who were free of recorded HF at landmark entry, greater within-person FIB-4 variability and persistent-high FIB-4 were associated with a higher subsequent HF hazard after explicit control for chronological age and other clinical characteristics. These associations remained present in analyses addressing documented pre-index cardiovascular disease. Worsening to high showed a more modest and less robust association, whereas improvement from high was not associated with HF.

Serial FIB-4 may represent a longitudinal marker of multisystem cardiometabolic burden, but its incremental predictive contribution was limited. The findings do not establish causality or support immediate clinical implementation. External validation and studies integrating serial hepatic, renal, cardiac, inflammatory, and imaging markers are required before serial FIB-4 can be incorporated into HF risk-assessment strategies.

## Data Availability

The data analyzed in this study is subject to the following licenses/restrictions: The data that support the findings of this study are available from Clinical Centre of the University of Debrecen, but restrictions apply to the availability of these data, which were used under license for the current study, and so are not publicly available. Data are however available from the authors upon reasonable request and with permission of Clinical Centre of the University of Debrecen, Hungary. Requests to access these datasets should be directed to nagy.attila@etk.unideb.hu.

## Supplementary material

Supplementary Table 1 Characteristics of the analytical cohort according to serial FIB-4 pattern.

Supplementary Table 2 Proportional-hazards diagnostics for the principal serial FIB-4 terms in the primary Cox models.

Supplementary Table 3 Audit of the joint strata used in the primary Cox models.

Supplementary Table 4 Sensitivity analyses addressing cardiovascular disease recorded before serial FIB-4 assessment.

Supplementary Table 5 Temporal predictive performance and calibration.

Supplementary Table 6 Characteristics of the temporal training and test cohorts.

Supplementary Table 7 Sensitivity analyses with HbA1c and recorded-diagnosis proxies.

Supplementary Table 8 Sensitivity analysis using a two-code definition of incident heart failure.

Supplementary Figure 1 Calibration of the baseline and extended models in the temporal test cohort at 1 and 2 years. Participants were grouped into quintiles according to predicted HF risk. Points show mean predicted risk and Kaplan–Meier observed risk within each group. The dashed diagonal line indicates ideal calibration. The baseline model included clinical covariates, landmark calendar time, and baseline FIB-4; the extended model additionally included serial FIB-4 features. Both models were evaluated in the later test cohort (n=1,617; 77 HF events).
