# Supplementary material for: Serial fibrosis-4 variability and longitudinal patterns are associated with incident heart failure in type 2 diabetes: a 24-month landmark cohort study
Source: Front Endocrinol (Lausanne). 2026 Oct 15;17:1963810. doi: 10.3389/fendo.2026.1963810 (PMC13716559; doi:10.3389/fendo.2026.1963810)
Supplement: Supplementary file 1 [file DataSheet1.docx]

**Table S1. Characteristics of the analytical cohort according to serial FIB-4 pattern**

| **Characteristic** | **Overall (N=8221)** | **Never high (N=7401)** | **Improving from high (N=252)** | **Worsening to high (N=277)** | **Persistent high (N=291)** |
| --- | --- | --- | --- | --- | --- |
| **Age, years** | **61.0 [52.0, 69.0]** | **60.0 [52.0, 69.0]** | **68.0 [61.0, 75.0]** | **68.0 [60.0, 75.0]** | **67.0 [60.0, 74.5]** |
| **Recorded diabetes duration, years** | **0.2 [0.0, 1.5]** | **0.2 [0.0, 1.5]** | **0.1 [0.0, 1.4]** | **0.4 [0.0, 1.8]** | **0.2 [0.0, 1.3]** |
| **Baseline FIB-4** | **1.12 [0.78, 1.59]** | **1.05 [0.75, 1.43]** | **3.23 [2.87, 3.91]** | **2.01 [1.48, 2.35]** | **4.26 [3.26, 6.12]** |
| **Last FIB-4 during exposure window** | **1.16 [0.81, 1.62]** | **1.09 [0.78, 1.47]** | **1.81 [1.24, 2.29]** | **3.22 [2.90, 3.79]** | **4.05 [3.19, 5.70]** |
| **Number of FIB-4 measurements** | **4 [3, 5]** | **4 [3, 5]** | **4 [3, 6]** | **4 [3, 6]** | **4 [3, 7]** |
| **FIB-4 variability, SD of log₂(FIB-4)** | **0.238 [0.145, 0.365]** | **0.225 [0.138, 0.341]** | **0.552 [0.312, 0.948]** | **0.467 [0.327, 0.725]** | **0.320 [0.192, 0.487]** |
| **FIB-4 slope, log₂ units/year** | **0.040 [-0.166, 0.257]** | **0.038 [-0.151, 0.240]** | **-0.588 [-1.150, -0.325]** | **0.600 [0.341, 0.911]** | **0.054 [-0.244, 0.281]** |
| **Fraction of exposure time with FIB-4 ≥2.67** | **0.000 [0.000, 0.000]** | **0.000 [0.000, 0.000]** | **0.555 [0.167, 0.722]** | **0.223 [0.111, 0.442]** | **1.000 [1.000, 1.000]** |
| **Female sex** | **4428 (53.9%)** | **4060 (54.9%)** | **113 (44.8%)** | **130 (46.9%)** | **125 (43.0%)** |
| **Hypertension** | **6204 (75.5%)** | **5540 (74.9%)** | **206 (81.7%)** | **228 (82.3%)** | **230 (79.0%)** |
| **Atrial fibrillation** | **443 (5.4%)** | **365 (4.9%)** | **23 (9.1%)** | **26 (9.4%)** | **29 (10.0%)** |
| **Chronic kidney disease** | **1221 (14.9%)** | **1082 (14.6%)** | **42 (16.7%)** | **53 (19.1%)** | **44 (15.1%)** |
| **Obesity** | **1270 (15.4%)** | **1180 (15.9%)** | **28 (11.1%)** | **29 (10.5%)** | **33 (11.3%)** |
| **Statin use** | **348 (4.2%)** | **318 (4.3%)** | **14 (5.6%)** | **13 (4.7%)** | **3 (1.0%)** |
| **RAAS inhibitor use** | **586 (7.1%)** | **531 (7.2%)** | **24 (9.5%)** | **18 (6.5%)** | **13 (4.5%)** |
| **Antiplatelet use** | **454 (5.5%)** | **400 (5.4%)** | **25 (9.9%)** | **19 (6.9%)** | **10 (3.4%)** |
| **SGLT2 inhibitor use** | **7 (0.1%)** | **5 (0.1%)** | **1 (0.4%)** | **0 (0.0%)** | **1 (0.3%)** |
| **GLP-1 receptor agonist use** | **97 (1.2%)** | **95 (1.3%)** | **1 (0.4%)** | **0 (0.0%)** | **1 (0.3%)** |

Values are median [interquartile range] or n (%). Serial patterns were defined from the first and last measurements in the 18-month exposure window using the prespecified FIB-4 threshold of 2.67. Abbreviations: FIB-4, Fibrosis-4 index; GLP-1, glucagon-like peptide-1; RAAS, renin–angiotensin–aldosterone system; SGLT2, sodium–glucose cotransporter-2.

**Table S2. Proportional-hazards diagnostics for the principal serial FIB-4 terms in the primary Cox models**

| **Model** | **Principal FIB-4 term** | **PH-test p-value** |
| --- | --- | --- |
| Variability model | Within-person FIB-4 variability | 0.667 |
| Pattern model | Worsening to high vs never high | 0.778 |
| Pattern model | Improving from high vs never high | 0.528 |
| Pattern model | Persistent high vs never high | 0.775 |

Proportional-hazards diagnostics were based on scaled Schoenfeld residuals with rank-transformed event time. No evidence against the proportional-hazards assumption was observed for any principal FIB-4 term (all p>0.05). PH, proportional hazards.

**Table S3. Audit of the joint strata used in the primary Cox models**

| **Joint stratum** | **Patients, n** | **HF events, n** | **Non-events, n** | **HF event proportion, %** |
| --- | --- | --- | --- | --- |
| **BASE_ALL__CAL_T3__HYP_0** | **541** | **11** | **530** | **2.0** |
| **BASE_T1__CAL_T1__HYP_0** | **321** | **56** | **265** | **17.4** |
| **BASE_T1__CAL_T1__HYP_1** | **654** | **200** | **454** | **30.6** |
| **BASE_T1__CAL_T2__HYP_0** | **342** | **24** | **318** | **7.0** |
| **BASE_T1__CAL_T2__HYP_1** | **622** | **113** | **509** | **18.2** |
| **BASE_T1__CAL_T3__HYP_1** | **513** | **38** | **475** | **7.4** |
| **BASE_T2__CAL_T1__HYP_0** | **257** | **73** | **184** | **28.4** |
| **BASE_T2__CAL_T1__HYP_1** | **784** | **299** | **485** | **38.1** |
| **BASE_T2__CAL_T2__HYP_0** | **189** | **35** | **154** | **18.5** |
| **BASE_T2__CAL_T2__HYP_1** | **709** | **181** | **528** | **25.5** |
| **BASE_T2__CAL_T3__HYP_1** | **666** | **58** | **608** | **8.7** |
| **BASE_T3__CAL_T1__HYP_0** | **225** | **87** | **138** | **38.7** |
| **BASE_T3__CAL_T1__HYP_1** | **795** | **326** | **469** | **41.0** |
| **BASE_T3__CAL_T2__HYP_0** | **142** | **33** | **109** | **23.2** |
| **BASE_T3__CAL_T2__HYP_1** | **750** | **202** | **548** | **26.9** |
| **BASE_T3__CAL_T3__HYP_1** | **711** | **75** | **636** | **10.5** |

Joint strata were constructed from baseline FIB-4 tertile, landmark calendar period tertile, and hypertension status. BASE_ALL denotes the prespecified collapse of baseline FIB-4 strata in the most recent calendar-period stratum among patients without recorded hypertension. Every final stratum contained at least five HF events and five non-events. Abbreviations: CAL, landmark calendar-period tertile; FIB-4, Fibrosis-4 index; HF, heart failure; HYP, hypertension status.

**Table S4. Sensitivity analyses addressing cardiovascular disease recorded before serial FIB-4 assessment**

| **Analysis** | **Model** | **Exposure** | **HR (95% CI)** | **P value** | **PH P value** | **Patients, n** | **HF events, n** | **Joint strata, n** |
| --- | --- | --- | --- | --- | --- | --- | --- | --- |
| **Full cohort adjusted for pre-index CVD** | **prior-CVD-adjusted variability** | **FIB-4 variability (SD of log2 FIB-4)** | **1.75 (1.45–2.12)** | **<0.001** | **0.483** | **8221** | **1811** | **16** |
| **Full cohort adjusted for pre-index CVD** | **prior-CVD-adjusted pattern** | **Persistent high vs never high** | **1.73 (1.35–2.22)** | **<0.001** | **0.766** | **8221** | **1811** | **16** |
| **Full cohort adjusted for pre-index CVD** | **prior-CVD-adjusted pattern** | **Worsening to high vs never high** | **1.27 (1.00–1.61)** | **0.047** | **0.738** | **8221** | **1811** | **16** |
| **Full cohort adjusted for pre-index CVD** | **prior-CVD-adjusted pattern** | **Improving from high vs never high** | **0.94 (0.69–1.27)** | **0.673** | **0.539** | **8221** | **1811** | **16** |
| **Sensitivity cohort without recorded pre-index CVD** | **CVD-free variability** | **FIB-4 variability (SD of log2 FIB-4)** | **1.90 (1.37–2.64)** | **<0.001** | **0.994** | **3762** | **605** | **16** |
| **Sensitivity cohort without recorded pre-index CVD** | **CVD-free pattern** | **Persistent high vs never high** | **2.34 (1.59–3.43)** | **<0.001** | **0.726** | **3762** | **605** | **16** |
| **Sensitivity cohort without recorded pre-index CVD** | **CVD-free pattern** | **Worsening to high vs never high** | **1.24 (0.77–2.02)** | **0.376** | **0.512** | **3762** | **605** | **16** |
| **Sensitivity cohort without recorded pre-index CVD** | **CVD-free pattern** | **Improving from high vs never high** | **0.83 (0.46–1.51)** | **0.542** | **0.530** | **3762** | **605** | **16** |

The full-cohort models additionally adjusted for recorded pre-index myocardial infarction, other ischemic heart disease, stroke, and peripheral arterial disease. The CVD-free sensitivity cohort excluded patients with any of these diagnoses recorded up to and including the index month. Model covariates and stratification otherwise followed the primary analyses. Abbreviations: CI, confidence interval; CVD, cardiovascular disease; FIB-4, Fibrosis-4 index; HF, heart failure; HR, hazard ratio; PH, proportional hazards.

**Table S5. Temporal predictive performance and calibration**

| Model | Horizon, years | Harrell C-index | IPCW Brier score | Mean predicted risk | KM observed risk | Calibration-in-the-large | Observed/expected ratio | Calibration slope (95% CI) |
| --- | --- | --- | --- | --- | --- | --- | --- | --- |
| Clinical model + baseline FIB-4 | 1 | 0.7081 | 0.0349 | 0.0383 | 0.0452 | −0.0069 | 1.1801 | 1.066 (0.734–1.398) |
| Clinical model + baseline FIB-4 | 2 | 0.7081 | 0.0390 | 0.0705 | 0.0733 | −0.0028 | 1.0402 | 1.066 (0.734–1.398) |
| Clinical model + baseline and serial FIB-4 | 1 | 0.7150 | 0.0349 | 0.0386 | 0.0452 | −0.0066 | 1.1719 | 1.009 (0.721–1.297) |
| Clinical model + baseline and serial FIB-4 | 2 | 0.7150 | 0.0389 | 0.0709 | 0.0733 | −0.0024 | 1.0333 | 1.009 (0.721–1.297) |

Models were fitted in the training cohort and evaluated in the temporally later test cohort without refitting (n=1,617; 77 HF events). Brier scores were estimated using inverse-probability-of-censoring weighting. Calibration-in-the-large is reported as mean predicted risk minus observed risk. Calibration slopes are shown with 95% confidence intervals. CI, confidence interval; HF, heart failure; IPCW, inverse-probability-of-censoring weighting; KM, Kaplan–Meier.

**Table S6. Characteristics of the temporal training and test cohorts**

| Characteristic | Training cohort | Test cohort | SMD |
| --- | --- | --- | --- |
| Patients, n | 6,604 | 1,617 | — |
| HF events, n | 1,734 | 77 | — |
| Landmark calendar year | 2013.29 ± 5.65; median 2013.00 (2009.00, 2018.00); missing 0 (0.0%) | 2024.17 ± 0.70; median 2024.00 (2024.00, 2025.00); missing 0 (0.0%) | −2.701 |
| Post-landmark follow-up, years | 7.40 ± 5.25; median 6.42 (2.92, 11.08); missing 0 (0.0%) | 1.10 ± 0.64; median 1.08 (0.58, 1.67); missing 0 (0.0%) | 1.683 |
| Age, years | 59.45 ± 13.55; median 61.00 (52.00, 69.00); missing 0 (0.0%) | 60.82 ± 13.80; median 63.00 (53.00, 71.00); missing 0 (0.0%) | −0.100 |
| Recorded diabetes duration, years | 1.42 ± 2.58; median 0.25 (0.00, 1.58); missing 0 (0.0%) | 1.73 ± 3.71; median 0.25 (0.00, 1.25); missing 0 (0.0%) | −0.097 |
| Baseline FIB-4 | 1.41 ± 1.53; median 1.12 (0.78, 1.57); missing 0 (0.0%) | 1.36 ± 1.02; median 1.11 (0.77, 1.65); missing 0 (0.0%) | 0.042 |
| FIB-4 measurement count | 4.40 ± 2.99; median 3.00 (2.00, 5.00); missing 0 (0.0%) | 4.85 ± 3.30; median 4.00 (3.00, 6.00); missing 0 (0.0%) | −0.142 |
| HbA1c, % | 7.46 ± 1.91; median 6.90 (6.10, 8.30); missing 3,297 (49.9%) | 7.03 ± 1.66; median 6.60 (5.99, 7.52); missing 710 (43.9%) | 0.237 |
| Female | 3,518 (53.3%); missing 0 (0.0%) | 910 (56.3%); missing 0 (0.0%) | −0.060 |
| Hypertension | 4,961 (75.1%); missing 0 (0.0%) | 1,243 (76.9%); missing 0 (0.0%) | −0.041 |
| Atrial fibrillation | 353 (5.3%); missing 0 (0.0%) | 90 (5.6%); missing 0 (0.0%) | −0.010 |
| Chronic kidney disease | 1,028 (15.6%); missing 0 (0.0%) | 193 (11.9%); missing 0 (0.0%) | 0.106 |
| Recorded obesity | 921 (13.9%); missing 0 (0.0%) | 349 (21.6%); missing 0 (0.0%) | −0.201 |
| Statin use | 251 (3.8%); missing 0 (0.0%) | 97 (6.0%); missing 0 (0.0%) | −0.102 |
| RAAS inhibitor use | 406 (6.1%); missing 0 | 180 (11.1%); missing 0 | -0.178 |
| Antiplatelet use | 377 (5.7%); missing 0 | 77 (4.8%); missing 0 | 0.043 |
| Recorded MASLD diagnosis | 366 (5.5%); missing 0 | 142 (8.8%); missing 0 | -0.126 |
| Tobacco-related diagnosis proxy | 11 (0.2%); missing 0 | 16 (1.0%); missing 0 | -0.109 |
| Alcohol-related diagnosis proxy | 250 (3.8%); missing 0 | 49 (3.0%); missing 0 | 0.042 |
| Serial pattern: improving from high | 194 (2.9%) | 58 (3.6%) | -0.037 |
| Serial pattern: never high | 5,957 (90.2%) | 1,444 (89.3%) | 0.03 |
| Serial pattern: persistent high | 233 (3.5%) | 58 (3.6%) | -0.003 |
| Serial pattern: worsening to high | 220 (3.3%) | 57 (3.5%) | -0.011 |
| NT-proBNP | 48.39 ± 86.79; median 12.80 (3.60, 53.20); missing 6571 | 599.12 ± 1476.28; median 122.46 (57.85, 378.00); missing 1596 | -0.527 |

Values are mean ± standard deviation; median (interquartile range); or n (%), as appropriate. Missing counts are shown for continuous variables where applicable. Standardized mean differences (SMDs) compare the training and test cohorts; negative values indicate lower values or prevalence in the training cohort. Post-landmark follow-up is shown descriptively and is not a baseline characteristic. The temporal test cohort was defined by landmark calendar period. HF, heart failure; MASLD, metabolic dysfunction-associated steatotic liver disease; RAAS, renin–angiotensin–aldosterone system.

**Table S7. Sensitivity analyses with HbA1c and recorded-diagnosis proxies**

| Model | FIB-4 term | HR (95% CI) | P value |
| --- | --- | --- | --- |
| HbA1c-adjusted | FIB-4 variability | 1.61 (1.33–1.95) | <0.001 |
| HbA1c-adjusted | Persistent high vs never high | 1.71 (1.36–2.15) | <0.001 |
| HbA1c-adjusted | Worsening to high vs never high | 1.27 (0.99–1.62) | 0.056 |
| HbA1c-adjusted | Improving from high vs never high | 0.92 (0.68–1.24) | 0.571 |
| HbA1c-adjusted + diagnosis proxies | FIB-4 variability | 1.57 (1.30–1.91) | <0.001 |
| HbA1c-adjusted + diagnosis proxies | Persistent high vs never high | 1.68 (1.34–2.11) | <0.001 |
| HbA1c-adjusted + diagnosis proxies | Worsening to high vs never high | 1.25 (0.97–1.59) | 0.079 |
| HbA1c-adjusted + diagnosis proxies | Improving from high vs never high | 0.91 (0.67–1.23) | 0.531 |

Each model included 8,221 participants and 1,811 HF events. The diagnosis-proxy models additionally included recorded MASLD, tobacco-use, and alcohol-related diagnoses. These variables are recorded-diagnosis proxies, not measured lifestyle exposures. CI, confidence interval; HF, heart failure; HR, hazard ratio; MASLD, metabolic dysfunction-associated steatotic liver disease. Missing HbA1c values were handled using multiple imputation by chained equations with 20 imputed datasets. Estimates were pooled using Rubin’s rules. Within-imputation variances were model-based; robust sandwich variance estimates were not used.

**Table S8. Sensitivity analysis using a two-code definition of incident heart failure**

| FIB-4 term | HR (95% CI) | P value |
| --- | --- | --- |
| FIB-4 variability | 1.37 (1.01–1.84) | 0.040 |
| Persistent high vs never high | 0.80 (0.50–1.26) | 0.329 |
| Worsening to high vs never high | 1.18 (0.82–1.70) | 0.361 |
| Improving from high vs never high | 0.89 (0.57–1.39) | 0.607 |

The sensitivity outcome required two distinct post-landmark months with an I50 code 7–365 days apart; the event date was the first code in the pair. The analysis included 8,221 participants and 833 HF events. Models used a ridge penalty of 0.0001. Confidence intervals and p-values were based on the approximate inverse penalized information matrix, not robust sandwich variance estimates. This stricter endpoint remains code-based and is not clinically adjudicated. CI, confidence interval; HF, heart failure; HR, hazard ratio.

**Figure S1. Calibration of the baseline and extended models in the temporal test cohort at 1 and 2 years. Participants were grouped into quintiles according to predicted HF risk. Points show mean predicted risk and Kaplan–Meier observed risk within each group. The dashed diagonal line indicates ideal calibration. The baseline model included clinical covariates, landmark calendar time, and baseline FIB-4; the extended model additionally included serial FIB-4 features. Both models were evaluated in the later test cohort (n=1,617; 77 HF events).**
